# Factors Influencing Changes in Food Preparation during the COVID-19 Pandemic and Associations with Food Intake among Japanese Adults

**DOI:** 10.3390/nu13113864

**Published:** 2021-10-28

**Authors:** Fumi Hayashi, Yukari Takemi

**Affiliations:** Faculty of Nutrition, Kagawa Nutrition University, 3-9-21 Chiyoda, Sakado-shi 3500288, Japan; takemi@eiyo.ac.jp

**Keywords:** cooking, COVID-19, diet, food preparation, adults, Japan

## Abstract

The primary aim of this study was to evaluate factors associated with changes in food-preparation practices during the Coronavirus Disease 2019 (COVID-19) pandemic in Japan and its associations to food-group intake. To examine this, a cross-sectional online survey was conducted in July 2020. Participants were 2285 adults aged 20–69 years who resided in any of 13 prefectures in Japan where specific COVID-19 regulations had been implemented. Self-reported changes in food-preparation practices when compared to the pre-COVID-19 pandemic period were measured as “increased” (24.6%), “decreased” (7.3%), and “no change” (68.1%), respectively. Stepwise logistic regression analyses indicated that participants who increased the time and effort for food preparation were younger in age, partially working remotely, experiencing reduced household income due to COVID-19, but highly concerned the importance of diet. On the other hand, participants whose household income decreased, and household economic status worsened, as well as those whose importance of diet deteriorated due to COVID-19 were more likely to decrease time and effort for cooking. Although the increased group were more likely to prepare meals with raw ingredients, the decreased group showed higher frequency of using takeout. These results indicated major determinants of changes in time spending on food preparation in consequence of COVID-19, and highlighted essential targets for future nutrition education.

## 1. Introduction

Coronavirus Disease 2019 (COVID-19) arrived in Japan in mid-January 2020, and, in February, the government declared the implementation of a “basic policy” for preventing the spread of infection that included activities such as information provision to the public, surveillance of the nationwide infection status, and assurance of quality medical care [1,2]. On 7 April 2020, the government declared an emergency in seven prefectures, and then, on 16 April, declared a nationwide emergency. At this time, 13 of the country’s prefectures, including the seven prefectures that were the targets of the initial emergency declaration, were categorized as “specific caution prefectures where efforts to prevent the spread of infection should be focused”. The nationwide state of emergency ended on 25 May 2020; each local government gradually reopened schools, and children slowly resumed school life after the long period of closure [1].

As a result of the emergency measures announced by the Japanese government, which encouraged people to stay at home and perform social distancing, the dietary behaviors of Japan’s adult population were impacted [3,4,5,6]. The increased time at home and time spent working from home brought favorable changes to people’s lifestyles, such as a rise in the level of attention devoted to cooking and meal preparation [3,4]. The Ministry of Agriculture, Forestry and Fisheries in Japan conducted a nationwide survey in December 2020 [3], asking respondents 13 questions regarding whether their current dietary habits differed to those before the COVID-19 outbreak. The question with the most “increased/expanded” responses concerned “eating at home” (35.5%), followed by “cooking at home” (26.5%), and “eating with family” (20.0%), respectively [3]. Lack of time is often considered a barrier to cooking, while a desire to improve one’s health and well-being through adopting a healthy diet is considered a facilitator [7,8]; this indicates that the increase in leisure time afforded by the emergency would result in dietary improvements. Several studies reported that people spent more time cooking or attending family meals during this period [4,9,10,11,12,13]. One study of Japanese adults found working from home during the COVID-19-related emergency period to be significantly associated with higher fruit, vegetable, and dairy product intake [4]. Another study with guardians from low-income households with children has reported significantly higher rates of having less time, psychological room, and financial position to prepare meals during the state of emergency during COVID-19 outbreak [5]. However, no previous research examined factors associated with changes in food-preparation behaviors in the context of the COVID-19 pandemic concerning Japanese adults and how such changes are associated with dietary intakes.

According to a study of Croatian adults reported that increased cooking frequency during confinement was associated with increased vegetable, legume, and fish and seafood consumption [9]. Among the US adults, cooking with fresh ingredients or using a recipe to make a meal were slightly correlated with fruit and vegetable intakes [13]. In Brazil, an Internet-based cross-sectional study showed that increased time spent on eating activities as well as increased frequency of cooking at home were shown to be associated with improved diet quality, but they did not investigate factors associated with changes in such behaviors [14]. Furthermore, this study did not examine the details of diet quality. According to a study conducted in Kosovo, on the other hand, a higher cooking frequency was positively associated with weight gain during the lockdown [15]. In Japan, one study reported increased frequency of self-made meals during the stage of emergency and an increase in frequency of consuming vegetables, beans, seaweed, fish, meat, dairy products, and snacks, and a decrease in frequency of alcohol consumption were observed, but this study only explored among diet app users [4]. In addition, the study did not examine associations between changes in the frequency of self-made meals and dietary intakes. Therefore, we know that the COVID-19 pandemic resulted in profound changes in food-preparation practices in some extent and it may influence on eating habits, potential factors associated with this modification remains unknown.

The aims of this study were to: (i) evaluate, among Japanese adults, factors associated with changes in the time and effort spent on food-preparation practices when compared to the pre-COVID-19 period, and (ii) examine whether different changes in the time and effort devoted to food-preparation practices are associated with differences in food-group intake.

## 2. Materials and Methods

### 2.1. Survey Methodology and Participants

This study is a cross-sectional study consisting of web-based survey conducted from 1–3 July 2020 to examine changes in dietary attitudes and habits of Japanese adults as a result of the COVID-19 outbreak. The sample comprised individuals aged 20–69 years who had resided in any of the 13 prefectures that had been designated “special alert prefectures” (where the Japanese government decreed that it was necessary to make concerted efforts to prevent the spread of infections) between April and May 2020. Voluntary participants were recruited via a consumer panel managed by Intage Inc. (Tokyo, Japan). As of December 2020, the consumer panel has 4.49 million registered monitors, with a gender ratio of 53% male and 47% female. We had a target sample size of 2000 individuals (1000 males and 1000 females; 400 people for each 10-year age group), by referring to the nationwide survey with nationally representative sample population on food and nutrition education undertaken by the Ministry of Agriculture, Forestry and Fisheries in October 2019 (final sample size: 1721 adults) [16]. The target age and gender ratios were determined based on the corresponding population ratios of each prefecture (these were determined using national census data [17]).

The first step was the performance of a screening test on approximately 10,000 potential participants; this was designed to include only individuals who had been involved in food preparation regularly during the COVID-19 pandemic. Participants were asked about frequency at the time of survey and chose from the items, “almost every day”, “4–5 times a week”, “2–3 times a week”, or “less than or equal to once a week”. Of the 4203 individuals who selected any of “almost every day”, “4–5 times a week”, or “2–3 times a week” for either cooking or grocery shopping and who were consequently invited to participate in the main survey. The research company collected the responses until the number of respondents reached the target sample size in accordance with the population composition of each prefecture [17], and 2389 participants were ultimately responded in this study. After excluding participants who did not reside in the 13 special alert prefectures from April to May 2020, those who did not correctly answer multiple questions, and those who did not cook for themselves, 2285 participants remained and were included in the analysis.

### 2.2. Measures

The survey collected data concerning sociodemographic characteristics, physical information, dietary attitudes and behaviors, and lifestyle variable, including changes in some variables between the pre-COVID-19 period and the time of the survey; further, food intake at the time of survey was also examined. Changes in home food-preparation practices since the pre-COVID-19 period were assessed through a single question (“Have you changed the amount of time and effort you spend on food preparation when compared to the period before the COVID-19 outbreak?”), for which the possible answers were “increased”, “decreased”, and “no change”, respectively.

#### 2.2.1. Sociodemographic Variables

The sociodemographic variables collected included demographic (age, gender, marital status, household status, and area of residence) and socioeconomic characteristics (employment status, job status, annual household income, household income change due to COVID-19, current household economic status, household economic status before the COVID-19 outbreak, household economic status change due to COVID-19, and highest educational qualification).

#### 2.2.2. Physical Variables

Physical variables collected included body mass index (BMI), changes in body weight when compared to the pre-COVID-19 period, disease diagnoses in the past year, and whether the respondents were subject to food restrictions for medical or religious reasons. BMI categories were determined using the participants’ self-reported heights and weights, and cut-offs were based on the Dietary Reference Intakes for Japanese 2020 [18]. These cut-offs were as follows—underweight: <18.5 kg/m^2^ for 20–49 years; <20.0 kg/m^2^ for 50–64 years; <21.5 kg/m^2^ for 65–69 years; normal: greater than or equal to underweight cut-offs and <25.0 kg/m^2^; and overweight/obese: ≥25.0 kg/m^2^. Whether the participants had been diagnosed with diseases such as hypertension, hyperlipidemia, diabetes, heart disease, cerebrovascular disease, or kidney disease within the past year was also evaluated.

#### 2.2.3. Dietary Attitudes and Behaviors

The variables collected concerning dietary attitudes and behaviors included dietary consciousness, cooking frequency, usual cooking practices, frequency of eating out, and frequency of eating takeout.

Dietary consciousness during the COVID-19 pandemic was measured using the Dietary Consciousness Scale [19]; a validated questionnaire that comprises 12 questions that are divided into two subscales: (1) importance of diet (seven items) and (2) precedence of diet when compared to other elements/activities (five items; Table S1). Total scores for the importance of diet range from 7 to 28, while those for the precedence of diet range from 5 to 20. For analyses, we grouped participants into two categories, “high score group” was above median and “low score group” was below median, by setting median as cut-off points for each subscale total score. To identify changes in dietary consciousness due to COVID-19, changes were obtained as responses for each item as follows—no change: 0 points, improved: +1 point, and worsening: −1 point. Thus, the total score for the change in the importance of diet ranged from −7 to +7 points, and those for the precedence of diet ranged from −5 to +5 points. After calculating the total score for each subscale, those who scored more than or equal to +1 point were grouped in “improved”, those who scored less than or equal to −1 point were grouped in “worsened”, and those who scored 0 point were grouped in “no change”.

Cooking frequency during the COVID-19 pandemic was measured using the question: “At present, how often do you cook for yourself or your family?”. There were four response options, ranging from “less than or equal to once a week” to “almost every day”. Usual cooking practices were measured with four response options, “prepare meals by cooking most things from raw ingredients”, “prepare meals by combining some commercial foods”, “prepare meals by combining many commercial foods”, and “prepare meals using commercial foods for everything”. Frequencies of eating out and eating takeout were both assessed using scales featuring seven responses, ranging from “none” to “more than twice a day”; further, responses were condensed into five categories for analyses.

#### 2.2.4. Lifestyle Variables

The lifestyle variables measured concerned physical activity and exercise habits and smoking status. Physical activity and exercise habits were assessed using the exercise standards and exercise guidelines for Japanese adults as a reference [20]. Participants reported whether their level of engagement in physical activity and exercise had “increased”, “decreased”, or showed “no change” when compared to the pre-COVID-19 period. Current smoking status was also evaluated, with ex-smokers and non-smokers being included in the “no” group.

#### 2.2.5. Food Intake

Food intake at the time of the survey was measured by examining frequency of consumption of 12 food groups that individuals are recommended to eat every day, and six food groups that individuals are recommended not to eat every day; these recommendations were based on the dietary guidelines for Japanese adults, as well as aspects of the Japanese diet that are recommended for preventing atherosclerotic cardiovascular disease [21,22,23]. Frequency of consumption of each food group was assessed using scales featuring four response options: “almost never eat”, “1–2 times a week”, “once every two days”, and “almost every day”; scores were calculated by awarding to each response 0.5, 1.5, 3.5, and 6.5 points, respectively [24]. After confirming the internal validity (Cronbach’s α = 0.818) for the recommended food groups, the total scores for each of these groups were calculated, and intergroup comparisons were performed. As the internal validity of the non-recommended food groups was not confirmed (Cronbach’s α = 0.416), we did not include the total scores in the analysis.

### 2.3. Ethics Approval

The survey was administered online by Intage Inc. (Tokyo, Japan). All participants were informed that the questionnaire was given for research purposes and their participation was completely voluntary. Since the data were collected anonymously using an online questionnaire, without including personal data, no written informed consent was required. The study protocol was approved by the ethics committee of Kagawa Nutrition University (Saitama, Japan; approval number: 291; approval date: 24 June 2020).

### 2.4. Statistical Analysis

Data were analyzed to evaluate which sociodemographic, physical, dietary, and lifestyle variables were associated with changes in the time and effort spent on food preparation since the pre-COVID-19 period. All analyses were performed using the statistical software package IBM SPSS Statistics version 27.0 (Armonk, NY, USA: IBM Corp).

First, chi-square tests and residual analyses were used to evaluate, among the three groups representing the different possible changes in home food-preparation practices (i.e., increased time and effort spent on food preparation, decreased time and effort, and no change, respectively), differences in sociodemographic, physical and lifestyle, and dietary behaviors and attitudes variables. We also performed chi-square tests and residual analyses to compare dietary and lifestyle variables among three groups by genders.

Second, multivariate logistic regression analyses using stepwise methods were performed to analyze factors that associated with changes in food-preparation practices and to calculate the odds ratio and 95% confidence interval (CI) of increased and decreased groups to the no-change group. Sociodemographic, physical, dietary and lifestyle variables were independent variables and selected using forward stepwise (Likelihood Ratio) selection procedure. Those whose employment status were others and those who did not know or did not want to answer job status, annual household income, highest educational qualification, as well as changes in body weight were considered to be missing value and excluded from analyses.

Third, a one-way analysis of variance was used to evaluate differences in food-group consumption frequency per week across the three food-preparation groups. Multiple comparisons among groups were performed using Bonferroni’s adjustment. Subsequently, the least square means were calculated and compared using a multiple one-way analysis of covariance, with adjustment for age group and gender.

## 3. Results

### 3.1. Changes in Food-Preparation Practices and Current Cooking Frequencies

Of the total 2285 participants, 24.6% (*n* = 563) reported increasing the time and effort they spent on food preparation when compared to the pre-COVID-19 period (hereafter, the “increased group”); 7.3% (*n* = 166) reported a decrease (hereafter, the “decreased group”) and 68.1% (*n* = 1556) reported no change (hereafter, “no-change group” Table 1). In terms of cooking frequency during the COVID-19 pandemic, participants of the increased group were more likely cooking almost every day (51.3%), or cooking 4–5 times a week (17.2%).

In the decreased group, significantly smaller number of participants who cooked almost every day. In terms of no-change group, participants who cooked less than or equal to once a week were significantly high among groups. However, in the analysis by gender, the percentage of women who cooked almost every day was significantly high in the “no-change” group (Table S2.).

### 3.2. Sociodemographic and Physical Variables

Table 2 shows a comparison of sociodemographic and physical variables among three groups. When comparing the three food-preparation groups in terms of sociodemographic variables, significant differences were found regarding gender, age group, employment status, job status, annual household income, changes in household income compared to the pre-COVID-19 period, household economic status before the COVID-19 pandemic, changes in household economic status when compared to the pre-COVID-19 period, and highest educational qualification. In terms of physical variables, there were significant differences regarding the variables of BMI category and having experienced a change in body weight when compared to the pre-COVID-19 period.

### 3.3. Dietary Attitudes, Behaviors, and Lifestyle Variables

In terms of dietary attitudes (Table 3), we studied dietary consciousness at the time of the study and its changes due to COVID-19. Members of the increased group were more likely to be high score group for both the importance (69.1%) as well as the precedence of diet (64.8%). In terms of the change in the importance of diet, significantly more improved participants (72.3%) were observed in the increased group, and more worsened participants (25.9%) were observed in the decreased group. In terms of the change in the precedence of diet, proportions of both improved and worsened participants were significantly higher in the increased and decreased groups. When examined by gender, the results were almost identical. However, in male, the proportions of participants who were in the high score for the precedence of diet was not significant (Table S2).

In terms of dietary behaviors, 44.6% (*n* = 251) of the participants in the increased group reported preparing most meals by cooking using raw ingredients. This association was present for both men and women (Table S3). Among the three groups, significant differences in the frequency of eating out or takeout were observed for both the overall sample and among males, wherein significant difference only in takeout among females. In the decreased group, significant more people were eating out or eating takeout at higher frequency (Table 3 and Table S3).

In terms of lifestyle variables, the increased group contained a significantly higher proportion of individuals who engaged in daily physical activity for one hour or longer (51.5%), who exercised for 30 min or longer more than twice a week (37.3%), and who reported increasing their engagement in physical activity or exercise when compared to the pre-COVID-19 period. Furthermore, both the increased (41.9%) and decreased (39.2%) groups showed significantly high proportions of individuals who decreased their engagement in physical activity or exercise.

### 3.4. Multivariate Logistic Regression Analyses of Factors Associated with Changes in Cooking Time and Effort

Table 4 shows the results of multivariate logistic regression analyses of the factors associated with changes in cooking time and effort compared to before COVID-19. Participants aged 20–49 years, partially working remotely, reduced household income due to COVID-19, importance of diet was high, changes in the importance or the precedence of diet were either improved or worsened, preparing meals using mainly raw ingredients, and changes in physical activity were either increased or decreased were more likely to be increased time and efforts spent on food preparation compared to no-change group. Participants with reduced household income as well as worsened household economic status were more likely to spend less time and efforts for food preparation compared to no-change group. In addition, participants with decreased body weight, changes in the precedence of diet either improved or worsened, higher frequency of using takeout, and decreased physical activity or exercise habits were more likely to decrease the time for food preparation. However, those whose scores of the precedence of diet were high and engaged in exercise greater or equal to 30 min or more than twice a week were less likely to be in the decreased group.

### 3.5. Food Intake and Desirable Eating Habits

In the adjusted model, significant changes since the pre-pandemic period were observed for almost all food groups for which daily consumption is recommended, except for potatoes (Table 5). After adjustment of age group and gender, the increased group showed the highest total score for the recommended food groups, significantly higher than the scores for the decreased and no-change groups. In terms of the not-recommended food groups, significant differences were observed regarding changes in consumption of alcoholic beverages, frozen meals, and instant products. In the decreased group, the frequency of consumption of frozen meals and instant products was significantly higher than that in the no-change group.

## 4. Discussion

The results of the present study showed that participants who, during the COVID-19 pandemic, increased the time and effort they devoted to food preparation were younger in age, partially working remotely, experiencing reduced household income due to COVID-19, but highly concerned the importance of diet. In addition, they prepared meals by cooking mostly raw ingredients or using only small amounts of commercial products. It has been reported that COVID-19 has had some positive effects on health-promoting behaviors such as home cooking [4,9,10,11,12,13], this is the first study to identify associated factors of changes in food-preparation behaviors among Japanese adults in the context of COVID-19 pandemic.

In the multivariate logistic regression analyses, the present study showed that sociodemographic factors such as being young adults and those who partially working remotely were found to be more likely to increase the time and effort for cooking during the COVID-19 pandemic. However, no significant association was found in gender. According to a previous study of 6325 participants sourced from across five countries, women and younger age groups are more likely to make healthy changes to their eating patterns during the COVID-19 pandemic [25]. In addition, women who feel that they have more leisure time and women who are required to work from home are more likely to make positive changes regarding diet planning and preparing healthy foods [26]. Gender is a stronger determinant of the time spent cooking than other sociodemographic variables, and women are more likely to engage in home cooking, mainly as a result of social and cultural aspects [27,28]. However, in the present study no gender differences were found in changes in home cooking practices. Although frequency of home cooking did not increase linearly with age [29], our finding indicates that those who were younger in age were more likely to increase their time and effort spent on food preparation. According to a study of Dutch adults, younger generations are more likely, when compared to older generations, to be influenced by COVID-19 lockdowns and to change their eating behaviors [30]. The National Health and Nutrition Survey conducted in 2019 has been reported that the younger the generation, the higher the frequency of eating out or the use of ready-to-eat meals in Japan [31]. Therefore, it is assumed that an increase in the frequency of cooking at home were observed among the younger generation who originally had more opportunities to eat out before COVID-19.

In terms of socioeconomic status, participants whose household income decreased due to COVID-19 were observed to be significantly more common in both the increased and decreased groups. Furthermore, those whose household economic status worsened were more likely to decrease their time and efforts for home cooking. Lower social class, particularly among women, has been found to be associated with spending more time cooking [27,32]. Although the present study did not observe a relationship between affluent status during the COVID-19 pandemic and changes in cooking practices, worsening household economic status might have led to an increase in the time and effort spent on food preparation because of an increased need to manage the household food budget. As the answers to the question regarding the change in time and effort devoted to preparing meals depended on the subjectivity of the participants, it is not possible to distinguish whether the time and burden associated with preparing meals actually increased. However, a previous study reported that due to COVID-19, the burden of preparing meals has increased for individuals of all economic conditions [5]. Therefore, the present study supports the results of previous studies. Further studies are necessary to evaluate whether the burden of cooking has lessened or worsened during the pandemic, and whether such changes have had a positive or negative effect on eating habits.

Participants whose perception for the importance of diet as well as the precedence of diet were high were more likely to increase home cooking during the COVID-19 pandemic but were less likely to decrease their time and effort for cooking. In addition, participants who had worsened their perception of the importance of diet due to COVID-19 were more likely to be in the decreased group. Although changes in dietary consciousness were associated both positively or negatively to changes in cooking time and efforts during COVID-19, this study showed that those in the increase group were more likely to prepare meals with raw ingredients, but those in the decrease group were more likely to buy takeout meals. Since cognitive factors such as knowledge, attitudes, and anticipated health consequences, are well known to affect food choice behaviors [33], high dietary consciousness of our participants might be associated with the change in food-preparation practices. Our findings support those of other studies that found associations between preparing food at home and diet quality. Participants who increased home cooking were more likely to have healthier food-consumption patterns, characterized by significantly higher total scores for recommended food groups, compared to decreased or no-change group. Although the concept of increasing the time and effort spent on cooking may vary widely between individuals, from full preparation of a meal from raw ingredients to simply increasing the frequency of cooking at home, the high proportion of people cooking from raw ingredients in the increased group may be associated with the relatively good eating habits observed for this group. Our findings accord with those of previous studies that found that increased cooking frequency during confinement has also been associated with an increase in vegetable, legume, and seafood consumption [9]. Furthermore, individuals with greater food-preparation skills, who spend longer time cooking, and who have a higher cooking frequency have known to have better-quality diets [34,35,36]. The guidelines of the Food and Agriculture Organization for the United Nations recommend consumption of plenty of fruit and vegetables and a diet rich in whole grains to maintain a healthy diet during the COVID-19 pandemic [37]. Combining this recommendation with the present findings indicates that to achieve and maintain a healthy diet, home cooking must be promoted.

On the other hand, participants who decreased the time and effort they devoted to cooking ate more frozen and instant food products than the other two groups (those who increased the time and effort they devoted to cooking at home and those who reported no change, respectively). Those who decreased their home cooking were also more likely to use takeout meals. Avoidance of cooking and increases in the consumption of convenience food products, such as ultra-processed foods (UPFs), has received attention to be associated with potentially negative health impacts [38,39,40,41,42,43]. High UPF consumption is associated with an increased risk of overweight/obesity, metabolic syndrome, reduced high-density lipoprotein cholesterol, as well asl increased risks of cardiovascular disease mortality, death from ischemic heart disease/cerebrovascular disease, and all-cause mortality [39,40,41]. In high-income countries, such as the United States or Canada, UPFs represent over 50% of total energy intake [42]; among Japanese adults this percentage is close to 40% [43]. Thus, a favorable change in the time spent engaging in home cooking practices during the COVID-19 pandemic might have positive influence on the diet of Japanese people.

Several limitations to this study should be considered when interpreting the findings. First, this was a cross-sectional study, sampling was not random, and the participants were limited to residents of prefectures where voluntary confinement was requested through special government alerts relating to the COVID-19 pandemic. In addition, the participants were limited to those who regularly engaged in food preparation. Although the participants were targeted by considering the age group and gender ratios of each prefecture (based on recent population census data [17]), it is not possible to generalize the present results to the entire population of Japan. Second, the participants were recruited from the panel of an Internet research company, and the survey was conducted on the Internet; web-based questionnaires with volunteer panels may feature recruitment and response biases [44]. To address such biases, in this study respondents who provided the same answer to all questions, including a reverse-scored item, were excluded from the analysis. Third, food-preparation behaviors, such as planning, shopping, and cooking, are complex to define and measure. In addition, frequency and time spent preparing food, self-estimated cooking skills and knowledge, enjoyment of cooking, ability to prepare meals from primary ingredients, and complex food-preparation techniques are important dimensions of food preparation; however, the only food-preparation changes measured in our analysis were time and effort. Fourth, the changes in food-preparation behaviors were self-reported, which creates a risk of both error and bias. Moreover, it is not possible to distinguish whether the increase in cooking time and effort was due to personal willingness to do so or unavoidable circumstances. Despite these limitations, however, the novel finding of our study was factors associated with changes in food-preparation behaviors during the COVID-19 pandemic among Japanese adults.

## 5. Conclusions

Our study showed that among Japanese adults, participants who, during the COVID-19 pandemic, increased the time and effort they devoted to food preparation were younger in age, partially working remotely, experiencing reduced household income due to COVID-19, but highly concerned the importance of diet. In addition, participants in the increased group had prepared meals by cooking mostly raw ingredients or using only small amounts of commercial products; thus, it was associated with better diet quality. On the other hand, those whose household income decreased and those whose household economic status worsened, as well as those whose importance of diet deteriorated due to COVID-19, were more likely to decrease their time to spend for cooking, which was related to eating more frozen meals and instant food products. As the COVID-19-related state of emergency in Japan is ongoing, we suggest that it is important to encourage people to spend time and effort preparing meals to maintain a healthy lifestyle. However, further longitudinal studies are needed to verify how increased time and effort spent preparing foods contributes to health or wellness.

## Figures and Tables

**Table 1:** Comparison of cooking frequency across the three groups that are based on changes in cooking time and efforts.

	Changes in Cooking Time and Effort Compared to before COVID-19						
	Increased (*n* = 563)		Decreased (*n* = 166)		No change (*n* = 1556)		
Cooking Frequency in COVID-19	*n*	%	*n*	%	*n*	%	*p* *^1^
almost every day	289 **	51.3	55 *	33.1	735	47.2	<0.001
4–5 times a week	97 **	17.2	24	14.5	167 *	10.7	
2–3 times a week	113	20.1	49 **	29.5	249 *	16.0	
less than or equal to once a week	64 *	11.4	38	22.9	405 **	26.0	

*^1^ Chi-square tests ** Adjusted residual ≥ 1.96, * Adjusted residual ≤ −1.96. Supplementary table (Table S2) is available by genders.

**Table 2:** Comparison of sociodemographic and physical variables across the three groups that are based on changes in cooking time and efforts (*n* = 2285).

		Changes in Cooking Time and Effort Compared to before COVID-19						
		Increased (*n* = 563)		Decreased (*n* = 166)		No Change (*n* = 1556)		
Sociodemographic Variables		*n*	%	*n*	%	*n*	%	*p* *^1^
Gender	Males	229 *	40.7	80	48.2	795 **	51.1	<0.001
	Females	334 **	59.3	86	51.8	761 *	48.9	
Age group, years	20–29	163 **	29.0	40	24.1	229 *	14.7	<0.001
	30–39	105	18.7	39	23.5	298	19.2	
	40–49	106	18.8	34	20.5	328	21.1	
	50–59	102	18.1	30	18.1	326	21.0	
	60–69	87 *	15.5	23 *	13.9	375 **	24.1	
Marital status	Unmarried	218	38.7	66	39.8	572	36.8	0.066
	Married	314	55.8	84	50.6	839	53.9	
	Divorced or widowed	31	5.5	16	9.6	145	9.3	
Household status	Living alone	144	25.6	47	28.3	357	22.9	0.173
	Couple	106	18.8	29	17.5	314	20.2	
	Couple with children	165	29.3	51	30.7	415	26.7	
	Others	148	26.3	39	23.5	470	30.2	
Employment status	Permanent employees	203	36.1	73 *	44.0	534	34.3	<0.001
	Contract employees	31	5.5	23 **	13.9	100	6.4	
	Part-time workers	88	15.6	27	16.3	264	17.0	
	Self-employed	39	6.9	5 *	3.0	132 **	8.5	
	Students	42 **	7.5	4	2.4	26 *	1.7	
	Housewives	114	20.2	2	12.7	282	18.1	
	Unemployed	45 *	8.0	13	7.8	215	13.8	
	Others	1	0.2	0	0.0	3	0.2	
Job status	Fully remote working	31	5.5	7	4.2	66	4.2	<0.001
	More remote working than working in the office	37 **	6.7	12	7.2	53 *	3.4	
	More working in the office than remote working	49 **	8.7	13	7.8	75 *	4.8	
	Fully working in the office	210	37.3	90 **	54.2	761 **	48.9	
	Currently not working	229 **	40.7	42 *	25.3	569	36.6	
	Don’t want to answer	6	1.1	2	1.2	32	2.1	
Annual household income, yen	<2,000,000	113	20.1	29	17.5	282	18.1	0.004
	2,000,000–4,000,000	107	19.0	44	26.5	311	20.0	
	4,000,000–6,000,000	119	21.1	30	18.1	271	17.4	
	≥6,000,000	149	26.5	41	24.7	375	24.1	
	Don’t know/don’t want to answer	75 *	13.3	22	13.3	317 **	20.4	
Household income change due to COVID-19	Increased	14	2.5	7 **	4.2	18 *	1.2	<0.001
	Reduced	272 **	48.3	73 **	44.0	450 *	28.9	
	No change	277 *	49.2	86 *	51.8	1088 **	69.9	
Household economic status	No affluence	244	43.3	75	45.2	682	43.8	0.282
	Neither	177	31.4	58	34.9	544	35.0	
	Affluence	142	25.2	33	19.9	330	21.2	
Household economic status before the COVID-19 outbreak	No affluence	185	32.9	60	36.1	571	36.7	0.001
	Neither	205 *	36.4	69	41.6	650	41.8	
	Affluence	173 **	30.7	37 **	22.3	335 *	21.5	
Household economic status change due to COVID-19	Improved	32	5.7	19 **	11.4	76 *	4.9	<0.001
	Worsen	137 **	24.3	39 **	23.5	210 *	13.5	
	No change	394 *	70.0	108 *	65.1	1270 **	81.6	
Highest educational qualification	Junior/high school	121 *	21.5	43	25.9	463 *	29.8	0.003
	Vocational school/college	132	23.4	41	24.7	357	22.9	
	University	280 **	49.7	74	44.6	645 *	41.5	
	Graduate school	27	4.8	6	3.6	61	3.9	
	Don’t want to answer	3 *	0.5	2	1.2	30 **	1.9	
**Physical variables**								
Body mass index category *^2^	Underweight	96	17.1	26	15.7	328 **	21.1	0.010
	Normal	366 **	65.0	106	63.9	893 *	57.4	
	Overweight/obese	75 *	13.3	26	15.7	281 **	18.1	
	Don’t want to answer	26	4.6	8	4.8	54	3.5	
Changes in body weight compared to before COVID-19	Increased	227 **	40.3	59	35.5	415 *	26.7	<0.001
	Decreased	75 **	13.3	31 **	18.7	130 *	8.4	
	No change	230 *	40.9	66 *	39.8	859 **	55.2	
	Don’t know	31 *	5.5	10	6.0	152 **	9.8	
Diagnosis of diseases *^3^	Yes	101	17.9	39	23.5	334	21.5	0.139
	No	462	82.1	127	76.5	1222	78.5	
Food restriction due to medical or religious reasons	Yes	25	4.4	11	6.6	74	4.8	0.503
	No	538	95.6	155	93.4	1482	95.2	

*^1^ Chi-square tests. *^2^ Cut-offs for body mass index categories differ among age groups according to the Dietary Reference Intakes: underweight (<18.5 kg/m^2^ for 20–49 years; <20.0 kg/m^2^ for 50–64 years; <21.5 kg/m^2^ for 65–69 years), normal (greater than or equal to underweight cut-offs and <25.0 kg/m^2^), overweight/obese is > 25.0 kg/m^2^. *^3^ Diagnosis of diseases such as hypertension, hyperlipidemia, diabetes, heart diseases, cerebrovascular diseases, and kidney diseases at health checkups within a year. ** Adjusted residual ≥ 1.96, * Adjusted residual ≤ −1.96.

**Table 3:** Comparison of dietary attitudes, behaviors and other lifestyle factors across the three groups that are based on changes in cooking time and efforts (*n* = 2285).

		Changes in Cooking Time and Effort Compared to before COVID-19						
		Increased (*n* = 563)		Decreased (*n* = 166)		No Change (*n* = 1556)		
Dietary Consciousness Scale		*n*	%	*n*	%	*n*	%	*p* *^1^
Importance of diet *^2^	High score group	389 **	69.1	79	47.6	723 *	46.5	<0.001
	Low score group	174 *	30.9	87	52.4	833 **	53.5	
Precedence of diet *^2^	High score group	365 **	64.8	73	44.4 *	947	60.9	<0.001
	Low score group	198 *	35.2	93	56.0 **	609	39.1	
Changes in the importance of diet due to COVID-19 *^3^	Improved	407 **	72.3	81	48.8	571 *	36.7	<0.001
	Worsened	33	5.9	43 **	25.9	72 *	4.6	
	No change	123 *	21.8	42 *	25.3	913 **	58.7	
Changes in the precedence of diet due to COVID-19 *^3^	Improved	260 **	46.2	68 **	41.0	365 *	23.5	<0.001
	Worsened	101 **	17.9	48 **	28.9	173 *	11.1	
	No change	202 *	35.9	50 *	30.1	1018 **	65.4	
**Dietary behaviors**								
Usual cooking practices	Prepare meals by cooking most things from raw ingredients	251 **	44.6	34 *	20.5	558	35.9	<0.001
	Prepare meals by combining some commercial foods	246	43.7	69	41.6	610	39.2	
	Prepare meals by combining many commercial foods	55 *	9.8	49 **	29.5	253	16.3	
	Prepare meals using commercial foods for everything	11 *	2.0	14	8.4	135 **	8.7	
Frequency of eating out	more than 4 times a week	36	6.4	22 **	13.3	81 *	5.2	<0.001
	2–3 times a week	51	9.1	27 **	16.3	131	8.4	
	once a week	71	12.6	25	15.1	195	12.5	
	less than once a week	225	40.0	53 *	31.9	637	40.9	
	none	180	32.0	39 *	23.5	512	32.9	
Frequency of takeout	more than 4 times a week	34	6.0	21 **	12.7	89	5.7	<0.001
	2–3 times a week	84	14.9	39 **	23.5	190 *	12.2	
	once a week	95	16.9	32	19.3	221 *	14.2	
	less than once a week	207	36.8	47 *	28.3	623 **	40.0	
	none	143	25.4	27 *	16.3	433 **	27.8	
**Lifestyle variables**								
Daily physical activity greater or equal to one hour	Yes	290 **	51.5	70	42.2	661 *	42.5	0.001
	No	273 *	48.5	96	57.8	895 **	57.5	
Exercise greater or equal to 30 minutes more than twice a week	Yes	210 **	37.3	43	25.9	487	31.3	0.006
	No	353 *	62.7	123	74.1	1069	68.7	
Changes in physical activity or exercise habits compared to before COVID-19	Increased	119 **	21.1	20	12.0	117 *	7.5	<0.001
	Decreased	236 **	41.9	65 **	39.2	363 *	23.3	
	No change	208 *	36.9	81 *	48.8	1076 **	69.2	
Smoking	Yes	108	19.2	39	23.5	291	18.7	0.329
	No	455	80.8	127	76.5	1265	81.3	

*^1^ Chi-square tests. *^2^ The importance diet as well as the precedence of diet were defined as above the median for the high score group and below the median for the low score group. The median scores were 20 for the importance of diet and 14 for the precedence of diet, respectively. *^3^ Changes in dietary consciousness were calculated as a total score based on answers for each item of the Dietary Consciousness Scale as follows: no change (0 point), improved (+1 point), and worsening (−1 point). After calculating the total score for each subscale, those who scored more than or equal to +1 point were grouped in “improved”, those who scored less than or equal to −1 point were grouped in “worsened”, and those who scored 0 point were grouped in “no change”. ** Adjusted residual ≥ 1.96, * Adjusted residual ≤ −1.96. Supplementary table (Table S3) is available by genders.

**Table 4:** Multivariate logistic regression analyses of the factors associated with change in cooking time and effort compared to before COVID-19, in comparison to “No-change” group.

		Increased (*n* = 445)				Decreased (*n* = 130)			
		OR *^1^	95%CI		*p*	OR *^1^	95%CI		*p*
**Sociodemographic variables**									
Gender	Males	ref.							
	Females	1.27	0.95	1.69	0.104				
Age group, years	20–29	3.41	2.26	5.14	<0.001				
	30–39	1.60	1.06	2.42	0.027				
	40–49	1.66	1.11	2.50	0.015				
	50–59	1.48	0.97	2.26	0.069				
	60–69	ref.							
Job status	Fully remote working	0.97	0.52	1.78	0.913				
	More remote working than working in the office	1.91	1.07	3.41	0.029				
	More working in the office than remote working	1.74	1.05	2.88	0.033				
	Fully working in the office	0.89	0.65	1.20	0.433				
	Currently not working	ref.							
Household income change due to COVID-19	Increased	1.72	0.66	4.46	0.264	3.04	0.87	10.68	0.083
	Reduced	1.95	1.49	2.54	<0.001	1.57	1.01	2.43	0.047
	No change	ref.							
Household economic status change due to COVID-19	Improved					1.59	0.95	2.67	0.080
	Worsen					2.25	1.11	4.54	0.025
	No change					ref.			
**Physical variables**									
Changes in body weight compared to before COVID-19	Increased					1.35	0.85	2.14	0.200
	Decreased					2.15	1.15	4.02	0.016
	No change					ref.			
**Dietary Consciousness**									
Importance of diet	High score group	1.56	1.17	2.06	0.002				
	Low score group	ref.							
Precedence of diet	High score group					0.60	0.38	0.93	0.023
	Low score group					ref.			
Change in the importance of diet due to COVID-19	Improved	2.71	1.95	3.76	<0.001	1.71	0.99	2.93	0.053
	Worsened	2.98	1.68	5.29	<0.001	6.52	3.35	12.70	<0.001
	No change	ref.				ref.			
Change in the precedence of diet due to COVID-19	Improved	1.95	1.43	2.66	<0.001	2.54	1.48	4.35	0.001
	Worsened	1.64	1.11	2.42	0.013	2.37	1.28	4.39	0.006
	No change	ref.				ref.			
**Dietary behaviors**									
Usual cooking practices	Prepare meals by cooking most things from raw ingredients	4.20	1.93	9.11	<0.001				
	Prepare meals by combining some commercial foods	3.85	1.80	8.27	0.001				
	Prepare meals by combining many commercial foods	2.01	0.88	4.59	0.097				
	Prepare meals using commercial foods for everything	ref.							
Frequency of takeout	more than 4 times a week					3.92	1.72	8.97	0.001
	2–3 times a week					3.40	1.73	6.71	<0.001
	once a week					2.33	1.19	4.58	0.014
	less than once a week					1.29	0.69	2.39	0.423
	none					ref.			
**Lifestyle variables**									
Exercise greater or equal to 30 min more than twice a week	Yes					0.50	0.30	0.82	0.006
	No					ref.			
Changes in physical activity or exercise habits compared to before COVID-19	Increased	2.95	2.03	4.28	<0.001	1.56	0.75	3.24	0.238
	Decreased	2.19	1.64	2.93	<0.001	1.78	1.13	2.79	0.013
	No change	ref.				ref.			

*^1^ OR (odds ratio) and 95%CI (confidence intervals) of “increased” (*n* = 445) and “decreased” (*n* = 130) groups were determined based on “no-change” group (*n* = 1105). Independent variables: sociodemographic variables (gender, age group, marital status, household status, employment status, job status, annual household income, household income change due to COVID-19, household economic status, household economic status before the COVID-19 outbreak, household economic status change due to COVID-19, highest educational qualification); physical variables (body mass index category, changes in body weight compared to before COVID-19, diagnosis of diseases, food restriction due to medical or religious reasons); Dietary Consciousness Scale (important of diet, precedence of diet, change in importance of diet due to COVID-19, change in precedence of diet due to COVID-19); dietary behaviors (usual cooking practices, frequency of eating out, frequency of takeout); lifestyle variables (daily physical activity greater or equal to one hour, exercise greater or equal to 30 min more than twice a week, changes in physical activity or exercise habits compared to before COVID-19, smoking). The logistic regression analysis was used to select independent variables using forward stepwise (Likelihood Ratio) selection procedure.

**Table 5:** Comparison of consumption frequency among the three groups that are based on changes in cooking time and efforts in unadjusted and adjusted models (*n* = 2285).

	Unadjusted				Adjusted			
	Increased (*n* = 563)	Decreased (*n* = 166)	No Change (*n* = 1556)	*p* *^1^	Increased (*n* = 563)	Decreased (*n* = 166)	No Change (*n* = 1556)	*p* *^2^
	Mean (SD)	Mean (SD)	Mean (SD)		LSM (SE)	LSM (SE)	LSM (SE)	
Food groups for which daily consumption is recommended *^3^								
Whole grains	1.72(2.09) ^a^	1.45 (1.79)	1.33(1.82) ^b^	<0.001	1.73 (0.08) ^a^	1.47 (0.15)	1.32(0.05) ^b^	<0.001
Fish and shellfish (excluding processed products)	2.12(1.52) ^a^	1.92 (1.51)	1.90(1.44) ^b^	0.011	2.15 (0.06) ^a^	1.96 (0.11)	1.89(0.04) ^b^	0.001
Lean meats (excluding processed products)	2.18(1.58) ^a^	1.78(1.40) ^b^	1.80(1.37) ^b^	<0.001	2.17 (0.06) ^a^	1.78(0.11) ^b^	1.80(0.04) ^b^	<0.001
Eggs	4.01(2.15) ^a^	3.36(2.17) ^b^	3.54(2.21) ^b^	<0.001	4.00 (0.09) ^a^	3.38(0.17) ^b^	3.55(0.66) ^b^	<0.001
Milk and dairy products (unsweetened)	4.13(2.50) ^a^	3.37(2.52) ^b^	3.88(2.59) ^a^	0.003	4.18 (0.11) ^a^	3.49(0.19) ^b^	3.84(0.06) ^b^	0.002
Soy and soy products	3.69(2.19) ^a^	2.84(1.98) ^b^	3.29(2.24) ^c^	<0.001	3.74 (0.09) ^a^	2.91(0.17) ^b^	3.26(0.06) ^b^	<0.001
Green and yellow vegetables	4.29(2.19) ^a^	3.34(2.25) ^b^	3.85(2.30) ^c^	<0.001	4.29 (0.09) ^a^	3.41(0.17) ^b^	3.84(0.06) ^b^	<0.001
Other vegetables	4.73(2.03) ^a^	3.48(2.24) ^b^	4.24(2.28) ^c^	<0.001	4.74 (0.09) ^a^	3.55(0.17)^b^	4.23 (0.06) ^c^	<0.001
Seaweeds	2.33 (1.84)	1.96 (1.66)	2.12 (1.87)	0.022	2.38 (0.08) ^a^	2.02 (0.14)	2.10(0.05) ^b^	0.005
Mushrooms	2.41(1.82) ^a^	1.93(1.45) ^b^	2.07(1.73) ^b^	<0.001	2.41 (0.07) ^a^	1.97(0.13) ^b^	2.06(0.04) ^b^	<0.001
Potatoes	1.67 (1.20)	1.58 (1.37)	1.53 (1.17)	0.059	1.67 (0.05)	1.59 (0.09)	1.53 (0.03)	0.061
Fruit (excluding processed products)	2.58(2.30) ^a^	2.30 (2.07)	2.27(2.19) ^b^	0.018	2.64 (0.09) ^a^	2.39 (0.17)	2.24(0.06) ^b^	0.001
Total Scores*^3^	35.85 (13.34) ^a^	29.32 (12.91) ^b^	31.81 (13.76) ^b^	<0.001	36.09 (0.56) ^a^	29.92 (1.02) ^b^	31.65 (0.33) ^b^	<0.001
Food groups for which daily consumption is not recommended *^4^								
Processed meat or fish products	2.06 (1.50)	1.95 (1.36)	2.03 (1.06)	0.711	2.07 (0.07)	1.95 (0.12)	2.03 (0.04)	0.674
Snack and desserts	3.16 (2.31)	2.94 (2.16)	3.05 (2.30)	0.468	3.12 (0.10)	2.95 (0.18)	3.06 (0.06)	0.686
Alcoholic beverages	2.09 (2.24)	2.30 (2.33)	2.14 (2.35)	0.592	2.29 (0.09)	2.41 (0.17)	2.05 (0.06)	0.022
Sweetened beverages	2.16 (2.24)	2.55 (2.27)	2.11 (2.26)	0.055	2.12 (0.10)	2.50 (0.17)	2.13 (0.06)	0.114
Frozen meals	1.70(1.43) ^a^	1.94(1.54) ^a^	1.46(1.28) ^b^	<0.001	1.69 (0.06) ^a^	1.91 (0.10) ^a,c^	1.47(0.03) ^b^	<0.001
Instant products	1.43(1.25) ^a^	1.82(1.59) ^b^	1.39(1.22) ^a^	<0.001	1.45 (0.05) ^a^	1.80(0.10) ^c^	1.38(0.03) ^a,b^	<0.001

*^1^ *p* values were calculated using ANOVA. Multiple comparison among groups were based on Bonferroni. There are significant differences between different alphabets. *^2^ *p* values were calculated using ANCOVA. Adjusted models include age group and gender. There are significant differences between different alphabets. *^3^ The average score was calculated by scoring 6.5 points for “almost every day”, 3.5 points for “once every two days”, 1.5 points for “1–2 times a week”, and 0.5 points for “almost never eat”. *^4^ After confirming internal validity (Cronbach’s α = 0.818) for recommended food groups, the total score was calculated. Internal validity of not-recommended food groups were not confirmed (Cronbach’s α = 0.416). SD: standard deviation, LSM: least squares mean, SE: standard error.

## Data Availability

Data sharing are not applicable.

## Supplementary Materials

The following are available online at https://www.mdpi.com/article/10.3390/nu13113864/s1, Table S1: Dietary Consciousness Scale, Table S2: Comparison of cooking frequency across the three groups that are based on changes in cooking time and efforts by genders, Table S3: Comparison of dietary attitudes, behaviors and other lifestyle factors across the three groups that are based on changes in cooking time and efforts and between genders.
