# Supplementary material for: Factors Influencing Changes in Food Preparation during the COVID-19 Pandemic and Associations with Food Intake among Japanese Adults
Source: Nutrients. 2021 Oct 28;13(11):3864. doi: 10.3390/nu13113864 (PMC8620480; doi:10.3390/nu13113864)
Supplement: Supplementary file 1 [file nutrients-13-03864-s001.zip › nutrients-1420604-supplementary.pdf]

Table S1. Dietary Consciousness Scale

No.	Scale items	Cronbach's alpha if item deleted
**Importance of diet** (Cronbach alpha = 0.838)		
1	I am interested in information regarding eating habits.	0.810
2	I have a higher interest in and awareness of healthy dietary habits than others.	0.804
3	I want to prioritize my eating habits.	0.814
4	I think I should devote a lot of time to my eating habits.	0.817
5	I think that I should invest money in my diet.	0.826
6	I endeavor to maintain a nutritional balance.	0.824
7	I want to know how to maintain nutritional balance.	0.814
**Precedence of diet** (Cronbach alpha = 0.734)		
1	Rather than preventing illnesses through eating habits, I think it is better to treat or think about illnesses only when they occur. #	0.699
2	I do not worry about my eating habits unless I or someone close to me experiences a diet-related problem. #	0.672
3	I value work or income more than having a healthy diet. #	0.702
4	I value play and hobbies more than my eating habits. #	0.694
5	I cannot afford to be mindful regarding my eating habits. #	0.672

Cronbach's alpha for the entire scale = 0.828

reverse items

Table S2. Comparison of cooking frequency across the three groups that are based on changes in cooking time and efforts by genders.

	Changes in cooking time and effort compared to before COVID-19						
	**Increased**		**Decreased**		**No change**		
**Cooking frequency in COVID-19**	**n**	**%**	**n**	**%**	**n**	**%**	p^{*1}
Males (n = 1,104)	(n=229)		(n=80)		(n=795)		
almost every day	62	27.1	12*	15.0	196	24.7	<0.001
4-5 times a week	43**	18.8	8	10.0	83*	10.4	
2-3 times a week	74**	32.3	29**	36.3	166*	20.9	
less than or equal to once a week	50*	21.8	31	38.8	350**	44.0	
Females (n = 1,181)	(n=334)		(n=86)		(n=761)		
almost every day	227	68.0	43*	50.0	539**	70.8	<0.001
4-5 times a week	54**	16.2	16	18.6	84*	11.0	
2-3 times a week	39	11.7	20**	23.3	83	10.9	
less than or equal to once a week	14*	4.2	7	8.1	55	7.2	

*1 Chi-square tests

**Adjusted residual ≥1.96

* Adjusted residual ≤-1.96

Table S3. Comparison of dietary attitudes, behaviors and other lifestyle factors across the three groups that are based on changes in cooking time and efforts and between genders.

		Male (n=1,104)							Female (n=1,181)						
		Increased (n=563)		Decreased (n=166)		No change (n=1,556)		*p*[*1]	Increased (n=563)		Decreased (n=166)		No change (n=1,556)		*p*[*1]
		n	%	n	%	n	%		n	%	n	%	n	%	
**Dietary Consciousness Scale**															
Importance of diet[*2]	High score group	155**	67.7	42	52.5	322*	40.5	<0.001	234**	70.1	37*	43.0	401*	52.7	<0.001
	Low score group	74*	32.3	38	47.5	473**	59.5		100*	29.9	49**	57.0	360**	47.3	
Precedence of diet[*2]	High score group	114	49.8	24*	30	419**	52.7	<0.001	251**	75.1	49*	57.0	528	69.4	0.003
	Low score group	115	50.2	56**	70	376*	47.3		83*	24.9	37**	43.0	233	30.6	
Changes in the importance of diet due to COVID-19[*3]	Improved	166**	72.5	36	45	255*	32.1	<0.001	241**	72.2	45	52.3	316**	41.5	<0.001
	Worsened	16	7	25**	31.3	49*	6.2		17	5.1	18**	20.9	23*	3.0	
	No change	47*	20.5	19*	23.8	491**	61.8		76*	22.8	23*	26.7	422**	55.5	
Changes in the precedence of diet due to COVID-19[*3]	Improved	87**	38	31**	38.8	181*	22.8	<0.001	173**	51.8	37**	43.0	184*	24.2	<0.001
	Worsened	57**	24.9	25**	31.3	99*	12.5		44	13.2	23**	26.7	74*	9.7	
	No change	85*	37.1	24*	30	515**	64.8		117*	35.0	25*	30.2	503**	66.1	
**Dietary behaviors**															
Usual cooking practices	Prepare meals by cooking most things from raw ingredients	72**	31.4	11*	13.8	182	22.9	<0.001	179**	53.6	23*	26.7	376	49.4	<0.001
	Prepare meals by combining some commercial foods	111**	48.5	31	38.8	315	39.6		135	40.4	38	44.2	295	38.8	
	Prepare meals by combining many commercial foods	36*	15.7	28**	35	187	23.5		19*	5.7	21**	24.4	66	8.7	
	Prepare meals by using commercial foods for everything	10*	4.4	10	12.5	111**	14.00		1*	0.3	4	4.7	24**	3.2	
Frequency of eating out	more than 4 times a week	26	11.4	16**	20.0	71*	8.9	<0.001	10	3.0	6	7.0	10	1.3	0.074
	2-3 times a week	32	14.0	21**	26.3	87*	10.9		19	5.7	6	7.0	44	5.8	
	once a week	27	11.8	13	16.3	105	13.2		44	13.2	12	14.0	90	11.8	
	less than once a week	85	37.1	20*	25.0	301	37.9		140	41.9	33	38.4	336	44.2	
	none	59	25.8	10*	12.5	231**	29.1		121	36.2	29	33.7	281	36.9	
Frequency of take-out	more than 4 times a week	24	10.5	16**	20.0	71*	8.9	<0.001	10	3.0	5	5.8	18	2.4	<0.001
	2-3 times a week	53**	23.1	18	22.5	124*	15.6		31	9.3	21**	24.4	66*	8.7	
	once a week	40	17.5	19	23.8	130	16.4		55	16.5	13	15.1	91*	12.0	
	less than once a week	66	28.8	19	23.8	273**	34.3		141	42.2	28*	32.6	350	46.0	
	none	46	20.1	8*	10.0	197**	24.8		97	29.0	19	22.1	236	31.0	
**Lifestyle variables**															
Daily physical activity greater or equal to one hour	Yes	132**	57.6	35	43.8	336*	42.3	<0.001	158	47.3	35	40.7	325	42.7	0.306
	No	97*	42.4	45	56.3	459**	57.7		176	52.7	51	59.3	436	57.3	
Exercise greater or equal to 30 minutes more than twice a week	Yes	116**	50.7	26	32.5	285*	35.8	<0.001	94	28.1	17	19.8	202	26.5	0.292
	No	113*	49.3	54	67.5	510**	64.2		240	71.9	69	80.2	559	73.5	

Changes in physical activity or exercise habits compared to before COVID-19	Increased	67**	29.3	12	15.0	51*	6.4	<0.001	52**	15.6	8*	9.3	66*	8.7	<0.001
	Decreased	81**	35.4	35**	43.8	172*	21.6		155**	46.4	30	34.9	191*	25.1	
	No change	81*	35.4	33*	41.3	572**	71.9		127*	38.0	48	55.8	504**	66.2	
Smoking	Yes	75	32.8	28	35.0	204*	25.7	0.036	33	9.9	11	12.8	87	11.4	0.658
	No	154	67.2	52	65.0	591**	74.3		301	90.1	75	87.2	674	88.6	

*1 Chi-square tests

*2 The importance diet as well as the precedence of diet were defined as above the median for the high score group and below the median for the low score group. The median scores were 20 for the importance of diet and 14 for the precedence of diet, respectively.

*3 Changes in the Dietary Consciousness were calculated as a total score based on answers for each item of the Dietary Consciousness Scale as follows: no change (0 point), improved (＋1 point), and worsening (－1 point). After calculating the total score for each subscale, those who scored more than or equal to +1 point were grouped in "improved", those who scored less than or equal to -1 point were grouped in "worsened", and those who scored 0 point were grouped in "no change."

**Adjusted residual ≥ 1.96

* Adjusted residual ≤ -1.96
